# Diagnostic Utility of Ultrasonography for Duodenal Ulcers in Pediatric Cases in Japan

**DOI:** 10.3389/fped.2019.00547

**Published:** 2020-01-20

**Authors:** Yasufumi Sakata, Hiroki Yasudo, Masashi Uchida, Mitsuru Saito, Yoshihiro Azuma, Shunji Hasegawa

**Affiliations:** ^1^Department of Pediatrics, Graduate School of Medicine, Yamaguchi University, Yamaguchi, Japan; ^2^Division of Pediatrics, Tokuyama Central Hospital, Yamaguchi, Japan; ^3^Division of Gastroenterology, Tokuyama Central Hospital, Yamaguchi, Japan

**Keywords:** upper gastroscopy, *Helicobacter pylori*, fecal occult blood test, computed tomography, antiulcer drug, HH sign, pediatric duodenal ulcer, ultrasound sonography

## Abstract

**Objective:** To evaluate the diagnostic utility of wall hypertrophy of the duodenal bulb with a hyperechoic lumen, designated as the “HH sign,” using ultrasound sonography (US) in pediatric duodenal ulcer (DU) patients.

**Study design:** We performed a US for five pediatric subjects diagnosed with DU by upper gastroscopy to determine the presence of the potentially diagnostic HH sign. The sonographic images were analyzed before and after DU treatment. Computed tomography was performed in three cases and fecal occult blood test (FOBT) in all five cases.

**Results:** Upper gastroscopy confirmed DU in all patients. While the HH sign was observed using US in four cases, with the DU located in the anterior bulb, the FOBT was positive in only one case. In these four cases, the HH sign diminished in response to treatment, as visualized by US. This was observed for both the initial as well as recurrent episodes. A mass-like region was observed in only one case, with the ulcer located in the proximity of the inferior duodenal wall.

**Conclusion:** The HH sign is useful for the follow-up of DU, and US may be a suitable modality for the follow-up. We believe that this diagnostic marker can aid in following up a greater number of DU cases.

## Introduction

Duodenal ulcer (DU), a major peptic ulcer, is defined as a disorder of mucosal defects in the duodenum. DU patients exhibit gastrointestinal symptoms such as abdominal pain, discomfort, nausea, and an episode of melena. *Helicobacter pylori* (*H. pylori*) infection is a major cause of DU and the eradication of *H. pylori* is essential to prevent the development of DU (1). In the duodenal bulb, anterior ulcers are common, with Kang et al. reporting that recurrent DUs were more likely to occur in the anterior region (2). While antiulcer drugs are the first choice of therapy, surgical and endoscopic therapies are additionally adopted in perforated or bleeding DU cases (3, 4). Although relatively common in adults, DU is rare in pediatric cases.

An upper gastroscopy is needed for diagnosis. However, it requires professional skills. Most patients undergo additional screening tests such as the fecal occult blood test (FOBT), X-ray, and/or computed tomography (CT); however, the latter two are associated with radiation exposure risks. It is reported that the FOBT is not useful for diagnosing DU (5).

Due to improvements in ultrasonic equipment, high resolution images can be obtained; therefore, several studies have used ultrasound sonography (US) to report the diagnostic utility of the hypertrophic wall of the duodenal bulb with its central echoic line in adult DU cases (6, 7). However, pediatric cases have received little attention (8). In this study, using US, we aimed to evaluate the hypertrophic wall of the duodenal bulb with a hyperechoic lumen (hereafter designated as the “HH sign”) as a potential diagnostic marker for DU in five pediatric patients.

## Methods

### Patients

Our study was a single center, retrospective case series. The subjects were five patients: three men and two women, aged between 11 and 14 years (mean age: 12.5 years), who were presented to the Tokuyama Central Hospital (Yamaguchi, Japan) between 2007 and 2017. Clinical features of these patients are shown in Table 1. This study was conducted in accordance with the recommendations of Institutional Review Board of Tokuyama Central Hospital. The protocol was approved by the Institutional Review Board of Tokuyama Central Hospital (K346-20190807). All subjects gave written informed consent in accordance with the Declaration of Helsinki.

**Table 1:** Clinical features of patients.

					**On admission**				
**Case no**.	Age^*^ (year)	**Sex**	**Symptom**	**Recurrent episodes**	**WBC (/μL)**	**Hb (g/dL)**	**CRP (g/dL)**	**Occult blood test**	**Findings of CT**
1	14	F	Abdominal pain	None	11970	14.5	0.56	Negative	Perforated DU
2	13	M	Upper abdominal pain	Two	7420	8.6	1.25	Negative	Non-perforated DU
3	12	F	Upper abdominal pain	Four	7370	13.0	0.17	Negative	Not done
4	11	M	Upper abdominal pain	None	6400	13.8	<0.02	Negative	Not done
5	13	M	Upper abdominal pain Dizziness	None	8500	9.2	<0.02	Positive	Unremarkable

* *Age at diagnosis; F, female; M, male; CT, computed tomography; WBC, white blood cells; Hb, hemoglobin; CRP, C-reactive protein; DU, duodenal ulcer*.

### Diagnostic Procedures

The US equipment used in this study included the Xario SSA-650A and Aplio400 from Canon Medical Systems Corporation (Tochigi, Japan), with convex (3.5, 6 MHz) and linear (8, 10 MHz) transducers. The liver, gallbladder, kidneys, pancreas, spleen, the gastrointestinal tract (gastroduodenal region, small intestine, colon, and rectum), and the aorta with its branches (such as the celiac and superior mesenteric arteries) were evaluated by US. CT was performed in three patients, and the FOBT was performed in all patients. DU was confirmed by upper gastroscopy in all cases. All patients underwent a proper treatment for DU, including the administration of proton pump inhibitors. *H. pylori* infection was confirmed in all cases. An antibiotic agent was administered to the patient with perforated DU, and an eradication therapy for *H. pylori* was adopted in all patients.

### Diagnostic Criteria

The duodenum was identified in the epigastric or right hypochondriac region. A normal image of the duodenal bulb shows three layers: the hyperechoic inner and outer layers and a hypoechoic middle layer (Supplementary Figure 1). The HH sign was determined to be the sonographic criterion of a DU, consisting of duodenal wall hypertrophy with thickness ≥5 mm, along with the central hyperechoic line.

## Results

### Case 1

**Figure 1:**
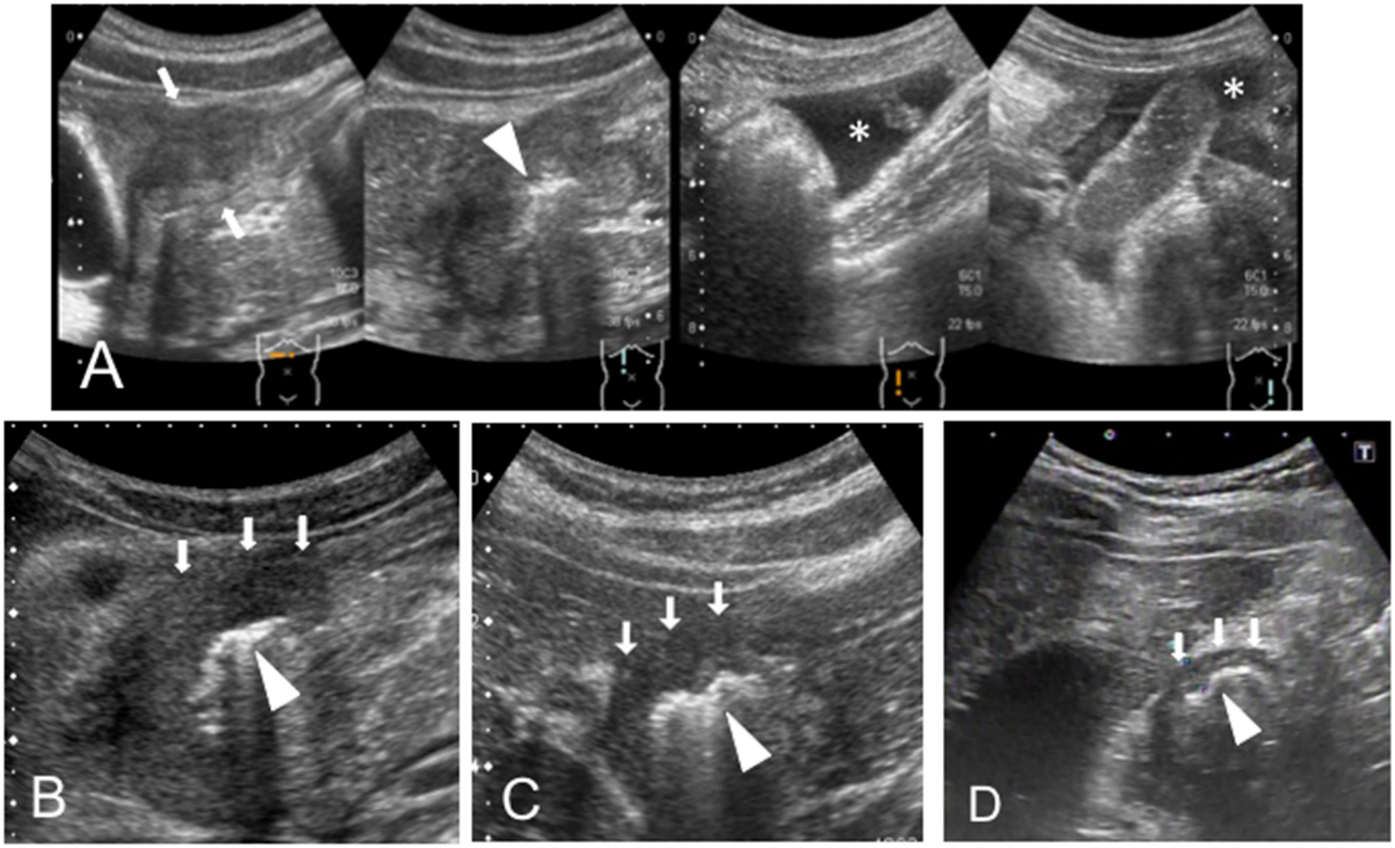
**(A)** Case 1, **(B)** Case 2, **(C)** Case 3, and **(D)** Case 4. The HH sign—extensive hyperplasia of the duodenal wall (white arrows) and the hyperechoic inner layer (white head-arrow)—was observed. The peritoneal fluid (*) is identified in case 1.

A 14-year-old girl was admitted to our hospital with sudden abdominal pain. Diffuse tenderness with abdominal guarding was observed on admission. The body height (BH) and body weight (BW) were 157 centimeters (cm) and 49.0 kilograms (kg) on admission, respectively. A blood test revealed slightly elevated counts of white blood cells (WBC: 12.0 ×10^9^/L) and C reactive protein (CRP: 0.56 mg/dL, reference range: ≤ 0.3 mg/dL), and the FOBT was negative (Table 1). We performed an abdominal sonography, which revealed peritoneal fluid accumulation and the HH sign in the duodenal bulb (Figure 1A). CT revealed edematous changes and wall defects in the duodenal bulb, which indicated a perforated DU. An antiulcer drug and antibiotic agent were administered, with concomitant fasting. Eight days after admission, we performed an endoscopy and identified ulceration in the duodenal bulb, on the basis of which, the patient was diagnosed with DU.

### Case 2

**Figure 2:**
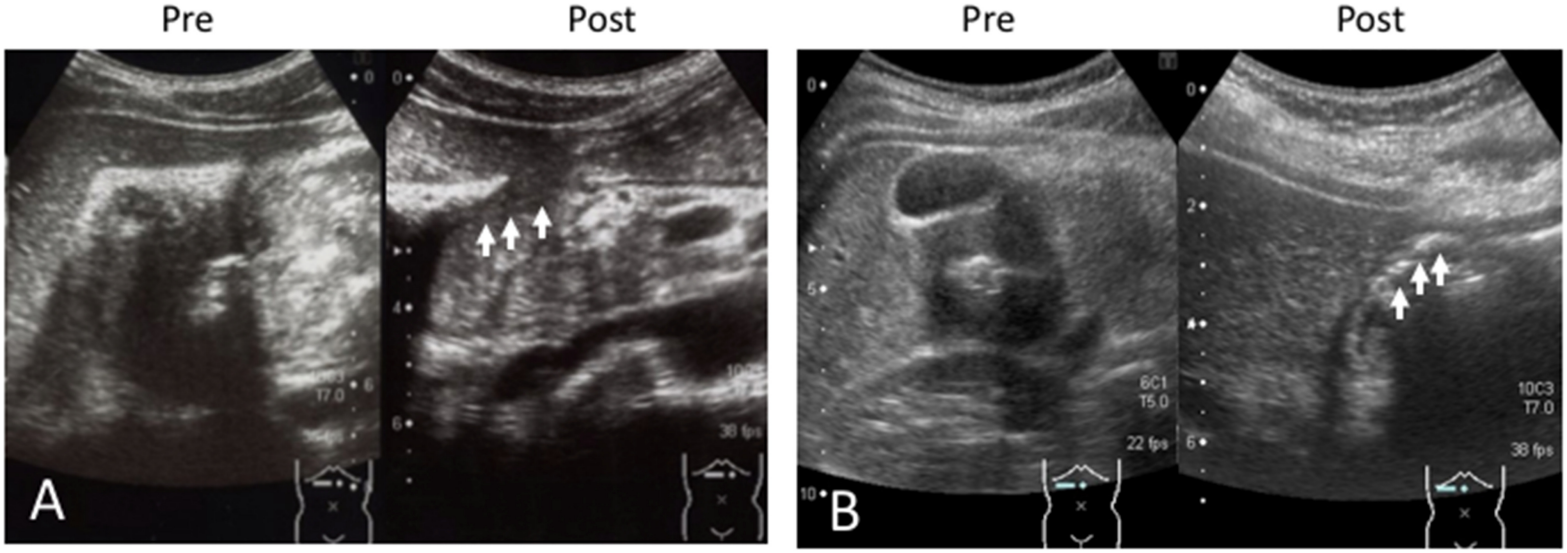
**(A)** Case 2 and **(B)** Case 3. The sonographic images in the recurrent episode are shown. The hypertrophic wall and echogenic line (HH sign) are shown in “Pre.” The HH sign diminished on treatment with the antiulcer drug (arrows) as shown in “Post.” Pre, pretreatment; Post, post-treatment with antiulcer agent.

A 13-year-old boy was presented to our hospital with upper abdominal pain lasting for 3 months. The BH and BW were 151.5 cm and 40.4 kg on admission, respectively. A blood test revealed anemia (hemoglobin 8.6 g/dL) with a slight elevation of CRP level (1.25 mg/dL), and the FOBT was negative (Table 1). An abdominal US scan revealed the presence of the HH sign (Figure 1B) and the absence of free air and ascites that usually indicate a perforated DU. CT revealed wall hyperplasia in the duodenal bulb, consistent with a DU. An endoscopy revealed a microhemorrhage in the anteriorly located DU. Five days after the initiation of the antiulcer drug, we performed a US and found that the HH sign had diminished (data not shown). He experienced two recurrent episodes, and in both, the HH sign, which was observed in the symptomatic period, diminished in the post-treatment period (Figure 2A).

### Case 3

A 12-year-old girl suffering from upper abdominal pain for several months was presented to our hospital. The BH and BW were 150 cm and 39.4 kg on admission, respectively. Her blood tests were normal (WBC 7.4 ×10^9^/L, hemoglobin 13.0 g/dL, CRP 0.17 mg/dL), and the FOBT was negative (Table 1). A US scan revealed the HH sign in the right epigastric region, consistent with her symptomatic site (Figure 1C). An endoscopy revealed DU on the anterior wall of the duodenal bulb. After the first episode, she experienced four recurrent episodes. In each, a pretreatment US scan revealed the HH sign, which disappeared in response to the treatment (Figure 2B).

### Case 4

An 11-year-old boy was hospitalized for epigastric pain. The BH and BW were 133 cm and 34.6 kg on admission, respectively. The thickness of the duodenal bulb wall was 5.4 mm (which slightly exceeded the 5 mm limit) and the hyperechoic line was clearly visible, consistent with the HH sign (Figure 1D). Blood tests were normal (WBC 6.4 ×10^9^/L, hemoglobin 13.8 g/dL, CRP <0.02 mg/dL), and the FOBT was negative (Table 1). We observed ulceration with highly mucosal edema, and the duodenal lumen was obstructed by the edematous change. The HH sign diminished after the initiation of the antiulcer drug (data not shown).

### Case 5

**Figure 3:**
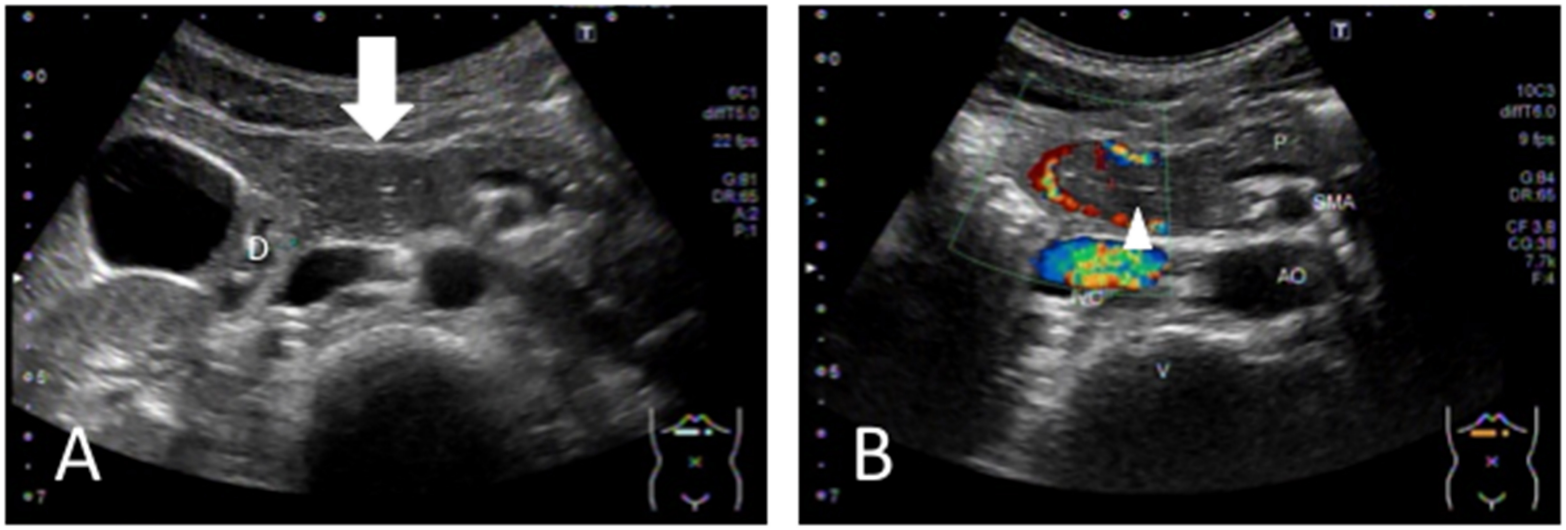
**(A)** The tumor-like region is identified (white arrow), which is located as part of the DU. **(B)** The blood vessel around the region and the central echogenic line (appearing to be the mucosa) are visualized. D, duodenum; P, pancreas; AO, aorta; IVC, inferior vena cava; SMA; superior mesenteric artery; V, vertebral column.

A 13-year-old boy was presented to our hospital with complaints of dizziness and upper abdominal pain. On admission, the BH and BW were 153 cm and 38.3 kg, respectively. Blood testing revealed anemia (hemoglobin 9.2 g/dL), and the FOBT was positive (Table 1). Since melena was observed on hospitalization, we performed a US, which revealed a mass-like region surrounded by a blood vessel (Figures 3A,B) that appeared to be connected to the duodenum. The HH sign was not observed. No remarkable findings were found in the CT image. In the endoscopy, the ulcer was identified at a site between the anterior and posterior bulb. A US performed 20 days after treatment revealed that the mass-like lesion disappeared.

## Discussion

Our study aimed to investigate whether the HH sign is a useful US finding for diagnosing pediatric DU cases. This sign was observed in four of the five pediatric DU cases. CT analysis performed in three cases revealed ulceration in two. Although the FOBT was performed in all cases, it was positive only in one. Our findings on US are compatible with previous reports in adult cases that showed that the HH sign was diagnostic for DU (6, 7). In previous reports, the presence of free air and variable amounts of free fluid were significant findings indicating perforated peptic ulcers (9–11). Free air is visualized as a reverberation, having a comet-tail appearance in the ultrasound (9, 10). Fam et al. (11) reported a case of a perforated DU with pneumoperitoneum, where air in the portal and systemic venous systems was confirmed by CT. In our study, in the patient with a perforated DU, there was no evidence of free air, but a relatively large amount of free fluid was revealed on US.

US, CT, FOBT, and upper gastroscopy are the commonly available screening tests for peptic ulcer, with some studies reporting the diagnostic utility of CT imaging for such ulcers in adult patients (12, 13). In our study, ulceration was observed in two of the three pediatric cases subjected to CT; however, Allen et al. (13) reported that the sensitivity of CT for non-perforated peptic ulcers was low and DUs were hardly identified compared to gastric ulcers. Further, radiation exposure is a matter of concern in children. Regarding acute radiation syndrome, it is reported that hematopoietic disorders are more likely to occur in children than in adults (14). Krille et al. (15) investigated the late effects of exposure to ionizing radiation from CT in a pediatric case, suggesting that this was a risk factor for a secondary cancer.

FOBT is commonly used to assess gastrointestinal diseases with bleeding. However, the Canadian Agency for Drugs and Technologies in Health states that its clinical effectiveness for diagnosis in patients with suspected gastrointestinal bleeding remains unknown (16). Nakama et al. (5) reported that the diagnostic sensitivity of FOBT was low in upper digestive tract diseases. This is in line with our observation, where that it was positive in only one case.

In general, endoscopy is mandatory for confirming DU, provides a local treatment in case of bleeding ulcer, and allows to perform biopsy samples for *H. pylori* diagnosis. Therefore, the interest of US could only be suggested in the patient with isolated epigastric pain showing no bleeding or growth retardation as endoscopy has to be performed irrespective of the US results in the cases with bleeding or other complication. Furthermore, the HH sign observed in recurrent episodes were reversible in case 2 and 3. Approximately 5 percent of DU patients is resistant to antiulcer drug and 20 percent of the patients relapses within 6 months after initial therapy (17, 18). US could be useful in the case of follow-up of DU patients with the recurrence risk because of its non-invasiveness.

The present study has several limitations. First, a US scan is dependent on the clinician's skill. Although a study reported that the sensitivity of US for the diagnosis of peptic ulcers in older children weighing >30 kg was low (8), in our study, DUs were identified using US in four out of the five pediatric patients weighing >30 kg. Additionally, factors such as bowel gas and obesity could negatively affect the examination. Second, the sample size was very small because pediatric DU patients are very rare and this study was a pilot study conducted in a single center. Further multi-center studies involving different regions are warranted to confirm the diagnostic utility of the HH sign in pediatric DU patients.

In conclusion, we showed that US identified the HH sign in four out of the five pediatric DU patients. The DU was located in the anterior bulb, a common site for these ulcers. Our findings suggest that this sign could be useful for the follow-up of pediatric DU.

## Data Availability Statement

All datasets generated for this study are included in the article/Supplementary Material.

## Ethics Statement

The studies involving human participants were reviewed and approved by the Institutional Review Board of Tokuyama Central Hospital. Written informed consent to participate in this study was provided by the participants' legal guardian/next of kin.

## Author Contributions

YS, HY, and SH were the principal investigators taking primary responsibility for the manuscript. YS, MU, and MS took responsibility for the diagnosis and data collection. HY, YA, and SH performed the clinical management with helpful discussion for the completion of the study.

### Conflict of Interest

The authors declare that the research was conducted in the absence of any commercial or financial relationships that could be construed as a potential conflict of interest.

## Supplementary Material

Supplementary Figure 1 The normal image of duodenal bulb in the healthy child. This image shows three layer (white arrowhead) and especially the thickness of middle layer is thin.

## Abbreviations

HH sign: hypertrophic wall of duodenal bulb with a hyperechoic lumen.

## References

[1] Ford AC Gurusamy KS Delaney B Forman D Moayyedi P Eradication therapy for peptic ulcer disease in *Helicobacter pylori*-positive people. Cochrane Database Syst Rev. (2006) 2:CD003840 10.1002/14651858.CD003840.pub4 PMC7163278 27092708

[2] Kang JY Guan R Tay HH Yap I Math MV Labrooy SJ. The site of recurrent duodenal ulcer. J Clin Gastroenterol. (1990) 12:621–3. 10.1097/00004836-199012000-00004 2266235

[3] Yan X Kuang H Zhu Z Wang H Yang J Duan X. Gastroduodenal perforation in the pediatric population: a retrospective analysis of 20 cases. Pediatr Surg Int. (2019) 35:473–7. 10.1007/s00383-018-4420-4 30448888

[4] Troland D Stanley A. Endotherapy of peptic ulcer bleeding. Gastrointest Endosc Clin N Am. (2018) 28:277–89. 10.1016/j.giec.2018.02.002 29933775

[5] Nakama H Kamijo N Fattah AS Zhang B. Immunologic detection of fecal occult blood from upper digestive tract diseases. Hepatogastroenterology. (1998) 45:752–4. 9684127

[6] Lim JH Lee DH Ko YT. Sonographic detection of duodenal ulcer. J Ultrasound Med. (1992) 11:91–4. 10.7863/jum.1992.11.3.91 1608082

[7] García Santos JM. Direct sonographic signs of acute duodenal ulcer. Abdom Imaging. (1999) 24:226–7. 10.1007/s002619900485 10227883

[8] Lee EJ Lee YJ Park JH. Usefulness of ultrasonography in the diagnosis of peptic ulcer disease in children. Pediatr Gastroenterol Hepatol Nutr. (2019) 22:57–62. 10.5223/pghn.2019.22.1.57 30671374 PMC6333589

[9] Patel SV Gopichandran TD. Ultrasound evidence of gas in the fissure for ligamentum teres: a sign of perforated duodenal ulcer. Br J Radiol. (1999) 72:901–2. 10.1259/bjr.72.861.10645199 10645199

[10] Fujii Y Asato M Taniguchi N Shigeta K Omoto K Itoh K. Sonographic diagnosis and successful nonoperative management of sealed perforated duodenal ulcer. J Clin Ultrasound. (2003) 31:55–8. 10.1002/jcu.10125 12478655

[11] Fam MNA Attia KME Khalil SMF. Case report: portal and systemic venous gas in a patient with perforated duodenal ulcer: CT findings. J Radiol Case Rep. (2014) 8:20–7. 10.3941/jrcr.v8i7.2070 25426236 PMC4242129

[12] Tonolini M Ierardi AM Bracchi E Magistrelli P Vella A Carrafiello G. Non-perforated peptic ulcer disease: multidetector CT findings, complications, and differential diagnosis. Insights Imaging. (2017) 8:455–69. 10.1007/s13244-017-0562-5 28677101 PMC5621988

[13] Allen BC Tirman P Tobben JP Evans JA Leyendecker JR Gastroduodenal ulcers on CT: forgotten, but not gone. Abdom Imaging. (2015) 40:19–25. 10.1007/s00261-014-0190-1 25015399

[14] Adams TG Sumner LE Casagrande R. Estimating risk of hematopoietic acute radiation syndrome in children. Health Phys. (2017) 113:452–7. 10.1097/HP.0000000000000720 28968347

[15] Krille L Zeeb H Jahnen A Mildenberger P Seidenbusch M Schneider K Computed topographies and cancer risk in children: a literature overview of CT practices, risk estimations and an epidemiologic cohort study proposal. Radiat Environ Biophys. (2012) 51:103–11. 10.1007/s00411-012-0405-1 22310909

[16] Urgent Non-screening Fecal Occult Blood Testing for Patients with Suspected Gastrointestinal Bleeding: A Review of Clinical Effectiveness and Guidelines. Ottawa, ON: Canadian Agency for Drugs and Technologies in Health (2017). Available online at: https://www.cadth.ca/urgent-non-screening-fecal-occult-blood-testing-patients-suspected-gastrointestinal-bleeding-review 28727402

[17] Hopkins RJ Girardi LS Turney EA. Relationship between Helicobacter pylori eradication and reduced duodenal and gastric ulcer recurrence: a review. Gastroenterology. (1996) 110:1244–52. 10.1053/gast.1996.v110.pm8613015 8613015

[18] Laine L Hopkins RJ Girardi LS. Has the impact of *Helicobacter pylori* therapy on ulcer recurrence in the United States been overstated? A meta-analysis of rigorously designed trials. Am J Gastroenterol. (1998) 93:1409–15. 10.1111/j.1572-0241.1998.452_a.x 9732917

