# Supplementary figures and images for: Diagnostic Utility of Ultrasonography for Duodenal Ulcers in Pediatric Cases in Japan
Source: Front Pediatr. 2020 Jan 20;7:547. doi: 10.3389/fped.2019.00547 (PMC6984193; doi:10.3389/fped.2019.00547)

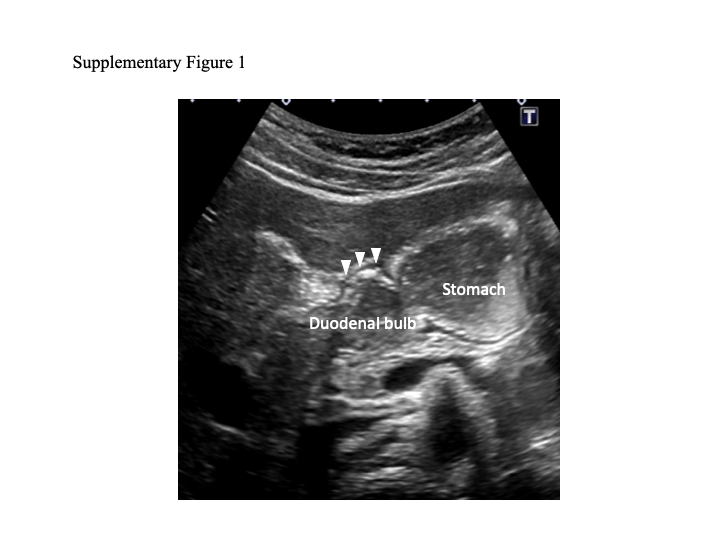

Supplement: Supplementary Figure 1 — The normal image of duodenal bulb in the healthy child. This image shows three layer (white arrowhead) and especially the thickness of middle layer is thin. [file Image_1.tiff]
